# Three divisions of the mouse caudal striatum differ in the proportions of dopamine D1 and D2 receptor-expressing cells, distribution of dopaminergic axons, and composition of cholinergic and GABAergic interneurons

**DOI:** 10.1007/s00429-019-01928-3

**Published:** 2019-08-02

**Authors:** Yuta Miyamoto, Issei Nagayoshi, Akinori Nishi, Takaichi Fukuda

**Affiliations:** 1 Department of Anatomy and Neurobiology, Graduate School of Medical Sciences, Kumamoto University, 1-1-1 Honjo, Chuo-ku, Kumamoto, 860-8556 Japan; 2 Department of Pharmacology, Kurume University School of Medicine, Kurume, 830-0011 Japan

**Keywords:** Striatum, Tri-laminar part, Dopamine receptor, Substance P, Enkephalin, Tyrosine hydroxylase

## Abstract

The greater part of the striatum is composed of striosomes and matrix compartments, but we recently demonstrated the presence of a region that has a distinct structural organization in the ventral half of the mouse caudal striatum (Miyamoto et al. in Brain Struct Funct 223:4275–4291, 2018). This region, termed the tri-laminar part based upon its differential immunoreactivities for substance P and enkephalin, consists of medial, intermediate, and lateral divisions. In this study, we quantitatively analyzed the distributions of both projection neurons and interneurons in each division using immunohistochemistry. Two types of projection neurons expressing either the dopamine D1 receptor (D1R) or D2 receptor (D2R) showed complementary distributions throughout the tri-laminar part, but the proportions significantly differed among the three divisions. The proportion of D1R-expressing neurons in the medial, intermediate, and lateral divisions was 88.6 ± 8.2% (651 cells from 3 mice), 14.7 ± 3.8% (1025 cells), and 49.3 ± 4.5% (873 cells), respectively. The intermediate division was further characterized by poor innervation of tyrosine hydroxylase immunoreactive axons. The numerical density of choline acetyltransferase immunoreactive neurons differed among the three divisions following the order from the medial to lateral divisions. In contrast, PV-positive somata were distributed throughout all three divisions at a constant density. Two types of GABAergic interneurons labeled for nitric oxide synthase and calretinin showed the highest cell density in the medial division. The present results characterize the three divisions of the mouse caudal striatum as distinct structures, which will facilitate studies of novel functional loops in the basal ganglia.

## Introduction

The striatum is the primary input structure of the basal ganglia and is abundantly innervated by axons originating from the cerebral cortex and thalamus. Principal neurons of the striatum consist of two types of GABAergic medium-sized spiny neurons (MSNs), the striatonigral MSNs (direct MSNs; dMSNs) and the striatopallidal MSNs (indirect MSNs; iMSNs). In rodents, axons of dMSNs project to the substantia nigra and entopeduncular nucleus with their terminals containing not only GABA but also substance P and dynorphin, whereas those of iMSNs innervate the globus pallidus with their terminals containing GABA and enkephalin (Harber and Watson 1983; Beckstead and Kersey 1985; Gerfen and Surmeier 2011). The activities of the MSNs are regulated by dopaminergic neurons residing in the substantia nigra pars compacta, and the two MSN types differ in their expression pattern of dopamine receptors; type 1 dopamine receptors (D1Rs) are predominant in dMSNs, and type 2 dopamine receptors (D2Rs) are predominant in iMSNs (Gerfen and Surmeier 2011). Regarding interneurons, cholinergic neurons (Bolam et al. 1984; Kubota and Kawaguchi 1993) and three distinct types of GABAergic neurons are known to exist in the striatum (Kita and Kitai 1988; Kita 1993; Kubota and Kawaguchi 1993; Kubota et al. 1993; Kawaguchi et al. 1995). The distribution pattern of each neuronal type tends to be biased according to both the compartmental and dorsolateral–ventromedial organizations inside the striatum (Kubota and Kawaguchi 1993; Fukuda 2009; Miyamoto et al. 2018).

The compartmentalization unique to the striatum is characterized by two complementary structures termed striosomes (patches) and matrix. These structures are clearly distinguishable using various chemical markers (Graybiel et al. 1981; Gerfen et al. 1985; Crittenden and Graybiel 2011). Many studies have aimed to explore both the morphological and functional differences between these two, apparently contrasting compartments, and this dichotomous view has greatly facilitated understanding of the striatum (Crittenden and Graybiel 2011). However, previous studies have also shown that striosome/matrix compartmentalization is not free from heterogeneity (Graybiel et al. 1981; Holt et al. 1997; Tajima and Fukuda 2013). Immunohistochemical labeling patterns of the mu-opioid receptor, substance P and enkephalin, all of which are representative markers of striosomes, show marked diversity that depends on the three-dimensional location inside the striatum (Tajima and Fukuda 2013; Miyamoto et al. 2018). The site-specific diversity of striosomes is also observed in the distributions of cannabinoid receptor-1 protein and mRNA (Davis et al. 2018). Moreover, the relative volume of striosomes in striatal tissue is high in the rostral striatum but decreases caudally, leading to disappearance of typical striosome/matrix compartmentalization in the ventral half of the most caudal striatum (Tajima and Fukuda 2013; Miyamoto et al. 2018). Instead, this region consists of three slab-like structures that can be identified as tri-laminar divisions in coronal sections, each of which can be distinguished based on SP and Enk immunoreactivities (Miyamoto et al. 2018). However, knowledge of cytoarchitecture in each division is greatly lacking.

The caudal striatum receives inputs from many sensory cortices, such as the visual, auditory, somatosensory and gustatory cortices (Jiang and Kim 2018). Primate studies have shown that the tail of the striatum, which corresponds to the caudal region of the rodent striatum, has a specific function for visual information that contributes to habitual behavior (Kim and Hikosaka 2013, 2015). In rodents, a recent study reported that caudal striatal neurons play an important role in auditory decision-making and provide a stable representation of sound in auditory tasks (Guo et al. 2018). Moreover, the caudal striatum, together with the lateral amygdaloid nucleus, receives inputs from the medial division of the medial geniculate nucleus (LeDoux et al. 1985) and contains neurons that directly respond to auditory signals (Bordi and LeDoux 1992). This suggests its involvement in the rapid phase of emotional behaviors to avoid risks in the environment, as in the amygdala (Romanski and LeDoux 1992). Involvement of the caudal striatum in the avoidance behavior has been further demonstrated by recent studies on midbrain dopamine neurons that project to the caudal striatum (Menegas et al. 2015, 2017, 2018). Therefore, the caudal striatum is one of intriguing regions to be studied with a special focus on, and here we analyzed the basic structure immunohistochemically.

In this study, distributions of both types of MSNs and four different classes of striatal interneurons were analyzed in the tri-laminar part of the mouse striatum using immunohistochemistry and a bias-free, stereology-based quantitative method. Results characterize and establish the three new divisions inside the ventral half of the caudal striatum.

## Materials and methods

### Tissue preparation

All experiments and animal procedures were performed according to the Guide for Care and Use of Laboratory Animals (National Institutes of Health Publications No. 80-23, revised 1986), and all of protocols were approved by the Institutional Animal Care and Use Committees at Kumamoto University and Kurume University. All efforts were made to minimize the number of animals used and their suffering.

Four male C57BL/6 J mice (21–26 g, 7–8 weeks old) and three transgenic mice tagged with D_1_R-DARPP-32-Flag/D_2_R-DARPP-32-Myc (Bateup et al. 2008; 25–30 g, 8–10 weeks old) were used for histological analysis. All animals were deeply anesthetized with sodium pentobarbital (100 mg/kg, i.p.) and perfused via the ascending aorta with 0.01 M phosphate-buffered saline (PBS, pH 7.4) followed by 50 ml of 4% paraformaldehyde (PFA) in 0.1 M phosphate buffer (PB, pH 7.4) at room temperature. Brains fixed with PFA were removed from the skull and stored overnight in the same fixative at 4 °C. The next day, the fixative was replaced with PBS containing 0.1% sodium azide.

### Immunohistochemistry

Serial 40-µm-thick coronal sections were cut using a vibrating microtome (TTK-3000, Dosaka) from brain blocks containing the entire tri-laminar part of the caudal striatum. After cryoprotection in 25% sucrose in PBS, sections placed on aluminum foil were rapidly frozen in the vapor of liquid N_2_, rapidly thawed in 25% sucrose in PBS, and then processed for triple-fluorescent immunohistochemistry, as previously described (Miyamoto and Fukuda 2015) using slight modifications. Primary and secondary antibodies used are listed in Tables 1 and 2, respectively. Briefly, sections were incubated in 5% normal donkey serum (Jackson ImmunoResearch) and 0.3% Triton-X in PBS overnight, followed by a mixture of rat anti-SP (1:500, Millipore), rabbit anti-Leu-Enk (1:500, Millipore), and sheep anti-TH (1:1000, Millipore) antibodies for 7 days at 20 °C. Subsequently, a mixture of Alexa 488-conjugated donkey anti-rat IgG (1:250, Jackson ImmunoResearch), Alexa 647-conjugated donkey anti-rabbit IgG (1:250, Jackson ImmunoResearch), and Rhodamine red-conjugated donkey anti-goat IgG (1:250, Jackson ImmunoResearch) were applied. The long incubation period with primary antibodies was essential to improve permeation of the antibodies into the deep portions of the 40-µm-thick sections to obtain confocal images of consistent and sufficient quality throughout the depth of the sections (Fukuda et al. 1998; Fukuda and Kosaka 2000).

**Table 1:** Primary antibodies used in this study

Antibody	Host species	Dilution	Source	Cat. #/lot #
SP	Rat	1:500	Millipore	MAB356/2857707
Leu-Enk	Rabbit	1:500	Millipore	AB5024/NMM1747747
TH	Sheep	1:1000	Millipore	AB1542/2896740
TH	Rabbit	1:1000	Millipore	AB152/NG1899903
PV	Mouse	1:5000	Swant	235/10-11 (F)
NOS	Sheep	1:10,000	Gift from Dr. Emson	
CR	Mouse	1:5000	Swant	6B3/010399
ChAT	Goat	1:1000	Millipore	AB144P/NG1915294
Flag	Mouse	1:200	Sigma-aldrich	F1804
c-Myc	Goat	1:2000	Novus biologicals	NB600-335

**Table 2:** Secondary antibodies used in this study

Antibody and fluorochrome	Conjugated fluorophore	Dilution	Source	Code. #/lot. #
Donkey anti-rat IgG	Alexa488	1:250	Jackson	712-545-150/131953
Donkey anti-mouse IgG	Alexa 488	1:250	Jackson	715-545-151/130814
Donkey anti-rabbit IgG	Alexa 647	1:250	Jackson	711-605-152/107850
Donkey anti-goat IgG	Rhodamine red	1:500	Jackson	705-295-147/133388

The second set of immunostaining experiments was prepared using sections from the transgenic mice. To investigate the distribution of D1R- and D2R-positive cells within the tri-laminar part of the caudal striatum, three sets of triple-immunostaining experiments were performed in 4 adjacent sections using a mixture of mouse anti-Flag (1:200, Sigma, F1804) and goat anti-c-Myc (1:2000, Novus biologicals, NB600-335) antibodies combined with one of the following antibodies: rat anti-SP (1:500, Millipore), rabbit anti-Met-Enk (1:500, Millipore) or anti-Leu-Enk (1:500, Millipore), along with rabbit anti-TH (1:1000, Millipore) antibodies. Sections were further incubated with the secondary antibodies listed in Table 2. Furthermore, several other sets of triple-immunostaining experiments were performed by combining the primary and secondary antibodies listed in Tables 1 and 2 to investigate the distribution of four types of interneurons: parvalbumin (PV)-, nitric oxide synthase (NOS)-, calretinin (CR)- and choline acetyltransferase (ChAT)-positive neurons.

Immunostained sections were mounted in Vectashield (Vector Laboratories) and examined using a confocal laser scanning light microscope (C2, Nikon), which was equipped with three single laser beams, 488, 543, and 633 nm in wavelength, and a filter set of BA 515/30, BA 590/50, and 650 LP. Control sections were prepared by omission of primary antibodies and by mismatching secondary antibodies. Both sets of controls provided only weak, nonspecific staining.

### Confocal laser scanning microscopy

Images for confocal laser scanning light microscopy (CLSM) were obtained using the 4 × (Plan Apo, N.A. = 0.2, Nikon), 20 × (Plan Fluor, N.A. = 0.5, Nikon), and 40 × (Plan Fluor, N.A. = 0.75, Nikon) objectives. The 4 × objective was used to visualize the entire striatum in a single frame in CLSM, whereas the 20 × and 40 × objectives were used to identify and analyze interneurons and MSNs with sufficient resolution, respectively. The size of each frame was 1024 × 1024 pixels, and images of optical slices were acquired from the section surface to the bottom at the preset optimal step size (one-third of full width at half maximum of *z* airy disk) and stored as stacked files for each frame using the three single laser beams alternately at each *z*-position of the stage to collect images of different fluorescence signals. The intensity of the signal in each pixel was recorded at 8 bits for each channel.

### Analysis

Immunoreactivity to the anti-Leu-Enk antibody was confirmed to be the same as that obtained using the anti-Met-Enk antibody, not only in the striatum but also in the globus pallidus, entopeduncular nucleus, and surrounding structures, such as the amygdala. Moreover, results with the anti-Leu-Enk antibody were consistent with those obtained in our previous studies using the anti-Met-Enk antibody (Miyamoto et al. 2018) and another antibody specific to both isoforms (Tajima and Fukuda 2013). Therefore, Enk immunoreactivity was collectively elucidated in the present study.

To determine the rostrocaudal extent of the tri-laminar part, CLSM images were acquired with a 4 × objective from serial coronal sections containing the entire tri-laminar part of the caudal striatum in mice, and sections were triple-labeled with SP, Enk, and TH. The medial and lateral divisions in the tri-laminar part were defined by a combination of SP and Enk immunoreactivities. The intermediate division was identified as the area between them. The distance of each section from the reference section that contained the center of the anterior commissure crossing the midline, which corresponded to the rostrocaudal coordinate of 0.13 mm rostral to the bregma in the standard atlas (Paxinos and Franklin 2013), was measured by multiplying the section thickness (40 µm) by the number of intervening sections.

Quantitative analysis of the distribution of D1R- and D2R-expressing cells inside the tri-laminar part was performed on every third section selected from serial sections covering the entire tri-laminar part of the caudal striatum, and CLSM images were acquired with a 20 × objective. The extent of each division in the tri-laminar part was identified by applying the contours traced from adjacent sections immunostained for either SP or Enk, and the number of somata was counted using Neurolucida. Cells were counted using a bias-free method disector (Sterio 1984; Miyamoto et al. 2018). Quantitative analysis of the distribution of PV-, NOS-, CR- and ChAT-positive neurons was also performed on every third section, and CLSM images were acquired with a 20 × objective. Contours of each division of the tri-laminar part were traced using adjacent sections triple immunostained for SP, Enk, and TH and were superimposed onto CLSM images of interneurons.

Gray values of fluorescent signals in CLSM images (4 × objective; no signal = 0, maximum level = 255) were measured along the line drawn mediolaterally in the middle of the tri-laminar part using the public domain program Image J (v.1.47). This line scan analysis was performed post hoc in the same confocal images that had been used for cell density analysis of interneurons. Each scanning line was divided into three divisions at the positions where inter-division borders, which had been traced manually on CLSM images before cell counting, crossed the scanning line. Then, the mean gray value in each division was calculated (horizontal dotted line in Fig. 3) and normalized using the highest gray value in each line as a unitary value of 1.

### Statistics

All statistical analyses were performed using the Tukey–Kramer test in the public program R, with *p *< 0.05 considered statistically significant.

## Results

### Characterization and extent of the tri-laminar part

**Fig. 1:**
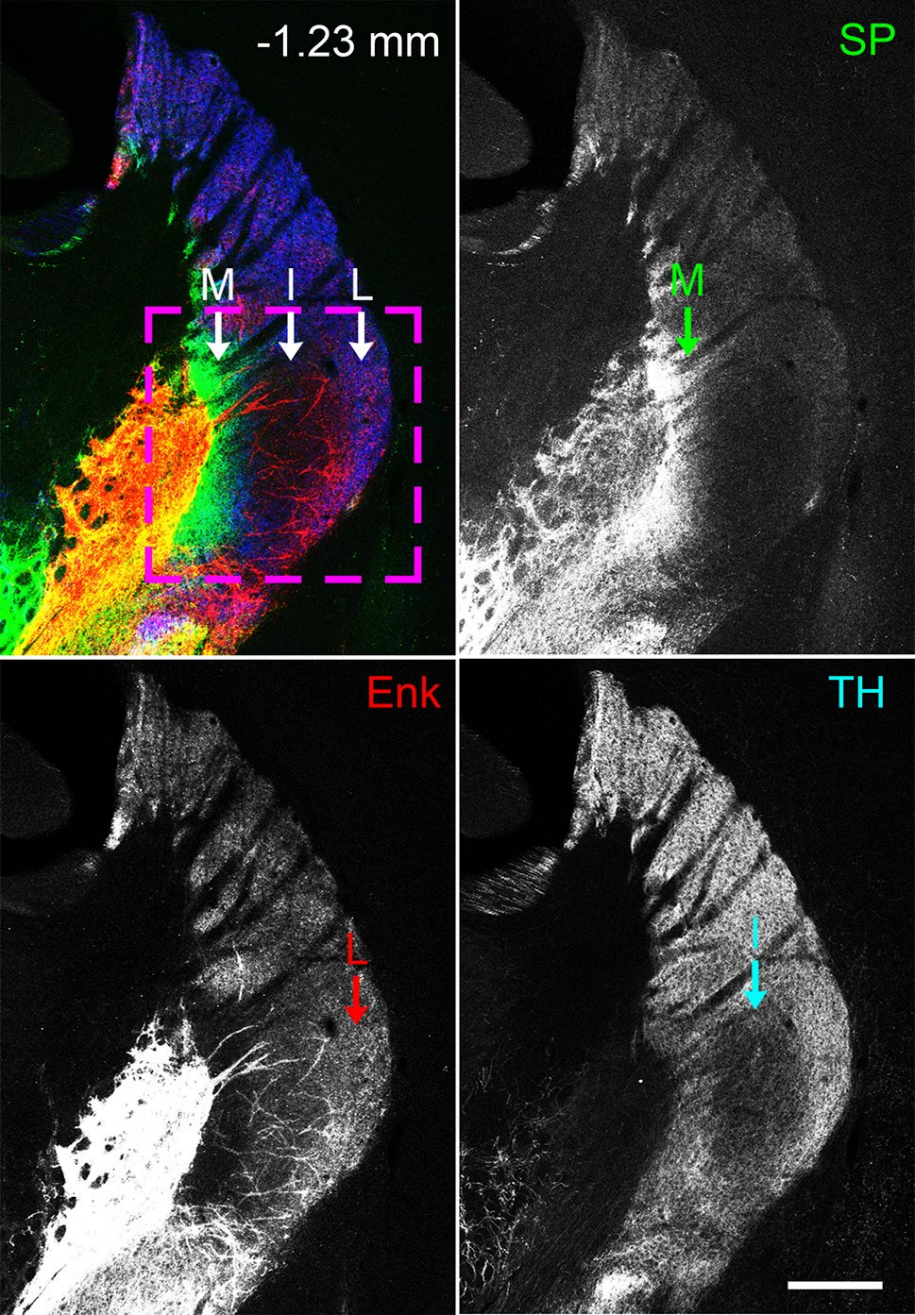
The tri-laminar part at the caudal striatum. Pseudocolored, CLSM images consisting of SP (green), Enk (red), and TH (blue) immunoreactivities in a coronal section located 1.23 mm caudal to the bregma. The rectangle indicates the tri-laminar part, where the medial division (M) is characterized by intense SP/faint Enk labeling, the intermediate division (I) by faint SP/weak Enk/weak TH labeling, and the lateral division (L) by faint SP/moderate Enk labeling. Scale bar 300 µm

Serial coronal sections containing the entire tri-laminar part of the caudal striatum were processed for triple immunohistochemistry using antibodies against SP, Enk, and TH (Fig. 1). Immunoreactivities for SP and Enk were observed in axon terminals but not in somata, because visualization of somata requires antibodies for the precursor forms, preprotachykinin for SP and preproenkephalin for Enk (Lee et al. 1997). TH immunoreactivity was detectable in both axons and axon terminals, whereas TH-positive somata that we observed in the substantia nigra pars compacta (SNpc) were not located in the tri-laminar part.

**Fig. 2:**
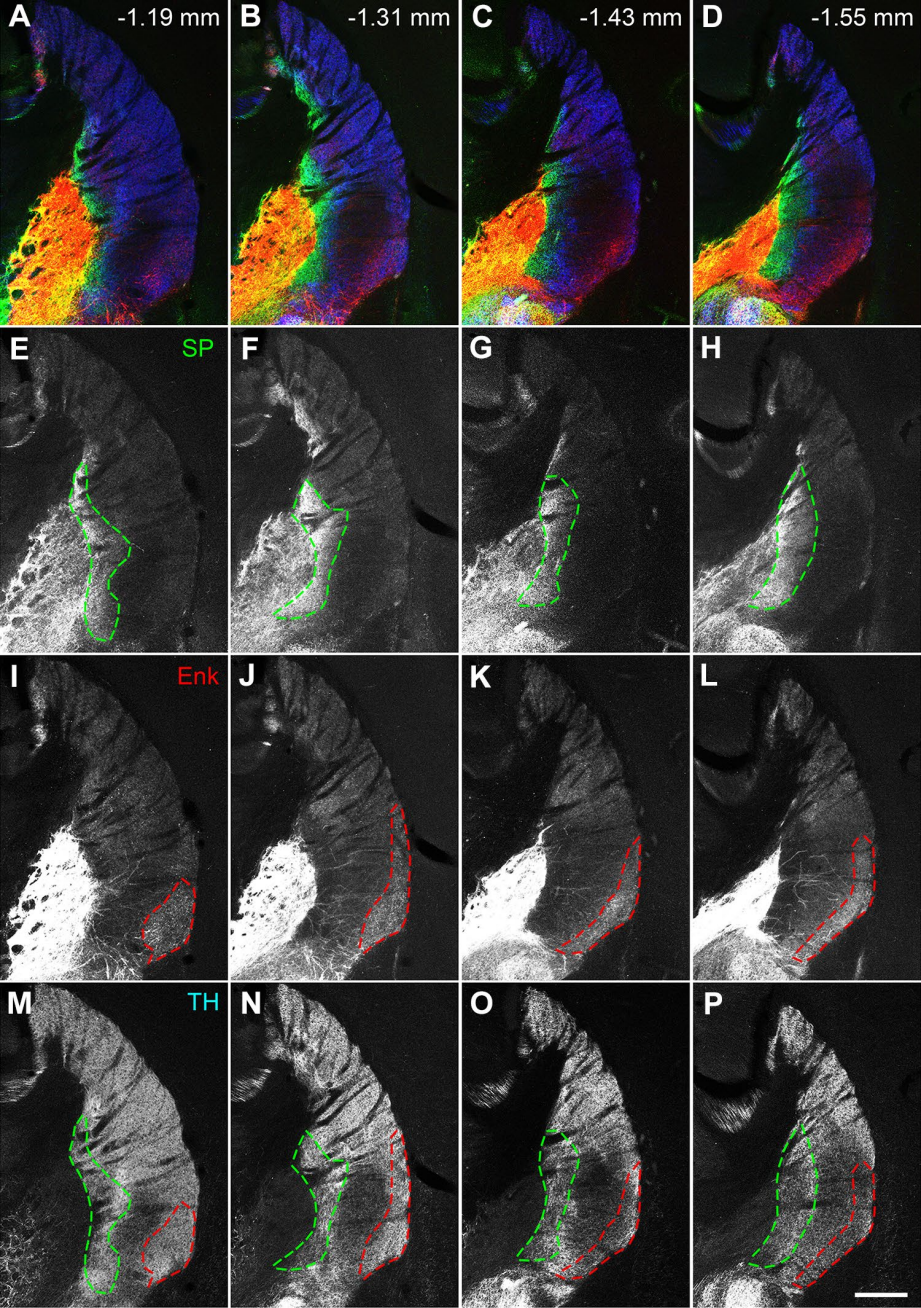
Extent of the tri-laminar part at four different positions along the rostrocaudal axis. Pseudocolor images (**a**–**d**) presenting, SP (green), Enk (red), and TH (blue) immunoreactivities are shown separately in **e**–**h**, **i**–**l**, and **m**–**p**, respectively. At the rostral levels (**a** and **b**), the intermediate division located between the medial (green dashed line) and lateral (red dashed line) divisions roughly corresponds to the area showing weak immunostaining for TH (**m** and **n**), the extent of which gradually expands toward the medial and lateral divisions at more caudal levels (**o** and **p**). Scale bar 300 µm

Immunostaining for SP and Enk was used to delineate each division of the tri-laminar part at different rostrocaudal levels (Figs. 1, 2). The medial division was characterized by highly intense labeling for SP and traces of Enk labeling, the lateral division by weak SP labeling and moderate labeling for Enk, and the intermediate division located between the medial and lateral divisions by faint labeling for both SP and Enk.

**Fig. 3:**
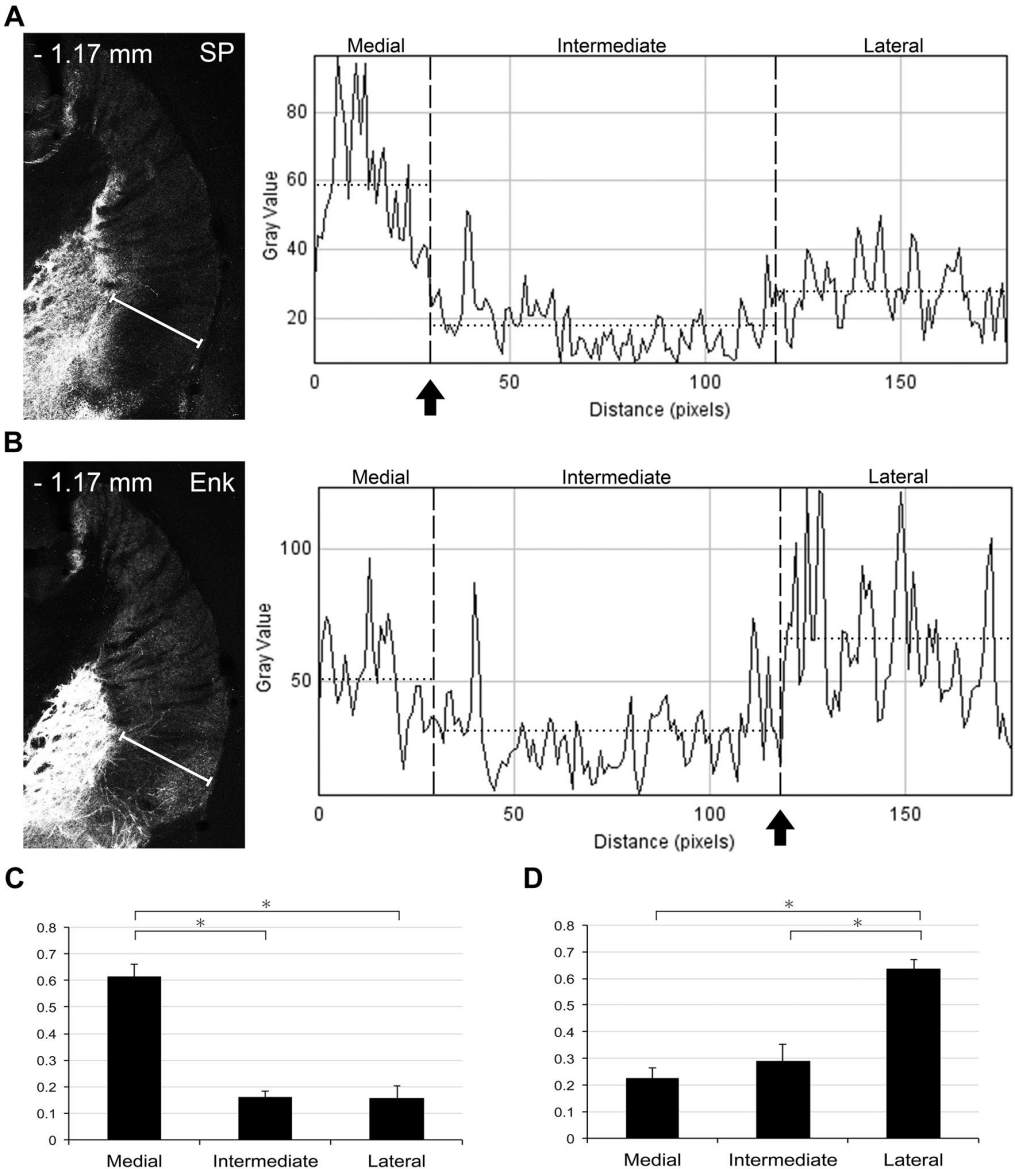
Line scan analysis of immunoreactivities for SP and Enk. Gray values of fluorescent signals in SP (**a**) and Enk (**b**) labeling were measured along the line drawn mediolaterally in the middle of the tri-laminar part. Arrows in the graph show the pre-determined position of the border between the medial and intermediate divisions in SP labeling (**a**) and that between the intermediate and lateral division in Enk labeling (**b**). Horizontal dotted line in each division indicates the mean gray value in that division, which was acquired at four different rostrocaudal positions in four animals, normalized and compared among three divisions for SP (**c**) and Enk (**d**). (Asterisks, *p* < 0.05, Tukey–Kramer test)

These patters were confirmed quantitatively by measuring gray values of fluorescent signals along the line drawn in the ventral half of the caudal striatum (Fig. 3). The graphs clearly indicate higher immunoreactivity for SP (Fig. 3a) and Enk (Fig. 3b) in the medial and lateral division, respectively. To analyze the differences statistically, mean gray value in each division (horizontal dotted line in Fig. 3a, b) was calculated in CLSM images that were acquired for analysis of interneuron density (Fig. 8) at four different rostrocaudal levels in four mice. The positions of inter-division borders (arrows in Fig. 3) were determined according to manually defined borders in CLSM images, and they coincided well with the positions where immunoreactivities changed abruptly from medial to intermediate division in SP labeling and from intermediate to lateral division in Enk labeling. Statistical analysis revealed that SP immunoreactivity in the medial division was significantly higher than that in both the intermediate (Tukey–Kramer test, *n* = 4 animals; *p* = 2 × 10^−7^) and lateral (*p* = 2 × 10^−7^) divisions (Fig. 3c). Likewise, Enk immunoreactivity in the lateral division was significantly higher than that in both the medial (*n* = 4 animals, *p* = 2.4 × 10^−6^) and intermediate (*p* = 1.0 × 10^−5^) divisions (Fig. 3d).

Based upon these observations, the extent of the tri-laminar part was found to range from 1.15 mm to 1.67 mm caudal to the bregma (Fig. 2). More caudally toward the caudal tip of the striatum (1.99 mm caudal to the bregma), the typical tri-laminar pattern was gradually lost and replaced by medial expansion of the Enk-rich division, which occupied most of the striatum and appeared to be in continuity with diffuse labeling in the amygdalostriatal transition area located dorsal to the central nucleus of the amygdala.

The intermediate division of the tri-laminar part was further characterized by weak immunoreactivity for TH as compared to surrounding regions (Figs. 1, 2). This was a remarkable feature, considering massive innervation of the striatum by dopaminergic axons from the SNpc (Matsuda et al. 2009) and relatively homogeneous staining in the rostral striatum of adult mice (Sato et al. 2008). The area showing weak TH immunoreactivity gradually expanded both medially and laterally at more caudal levels, where TH labeling was weak throughout the ventral half of the caudal striatum (Fig. 2p).

### Distribution of D1R- and D2R-expressing neurons

**Fig. 4:**
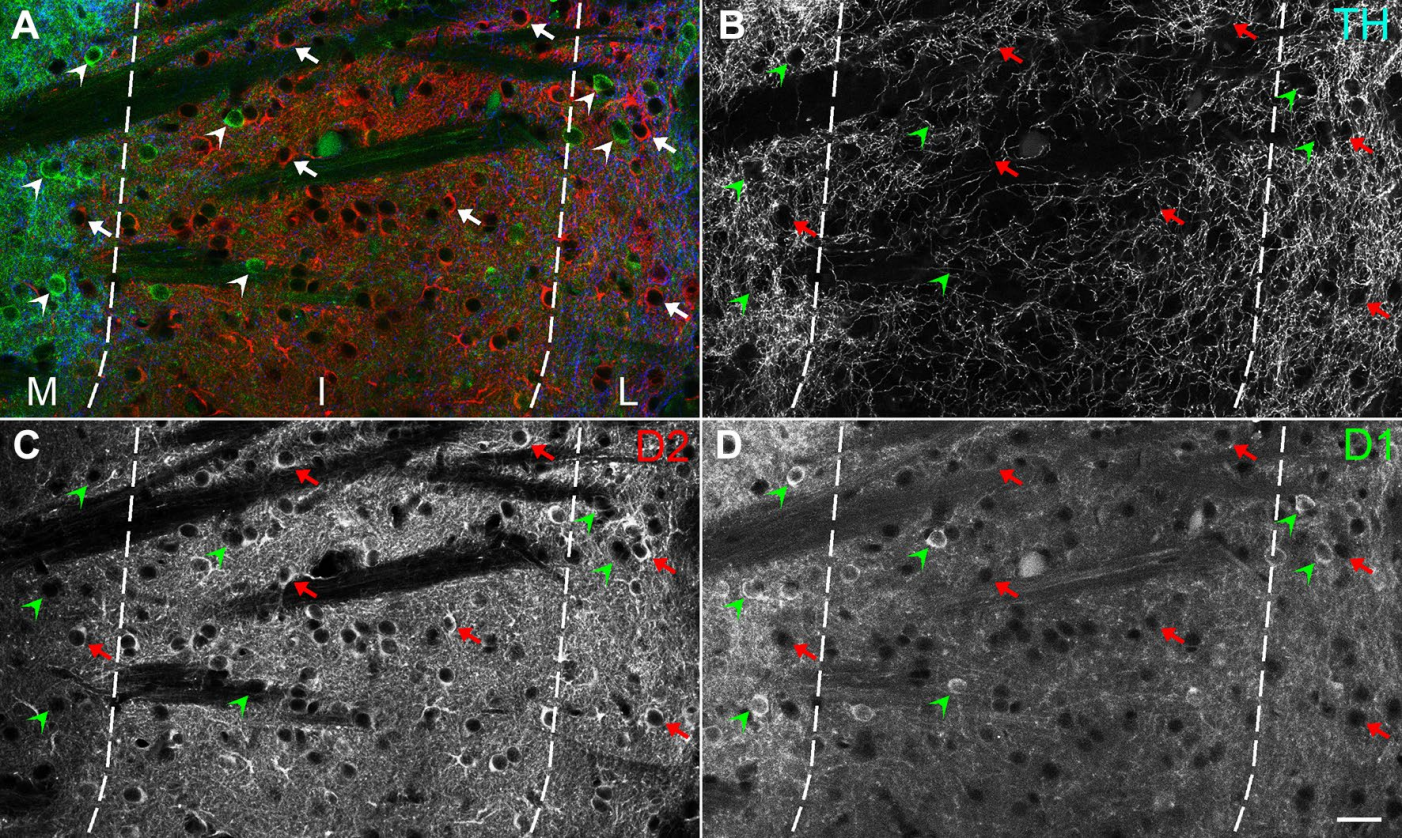
Distribution of dopamine D1R- and D2R-expressing cells in the tri-laminar part. The pseudocolor image in **a** consists of TH (blue), D2R (red), and D1R (green) immunoreactivities, which are shown separately in **b**–**d**, respectively. D1R- and D2R-expressing cells are indicated by arrowheads and arrows, respectively. The vast majority of cells in the medial division (M) express D1 receptors, whereas D2R-expressing cells outnumber D1R-expressing cells in the intermediate division (I). In the lateral division (L), both D1R- and D2R-expressing cells are distributed with similar densities. Scale bar 25 µm

**Fig. 5:**
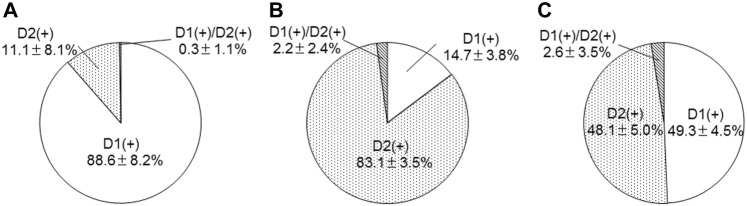
Proportions of D1R- and D2R-expressing cells in the tri-laminar part. Cells detectable with the two immunoreactivities are classified into three groups, those labeled for D1R only, D2R only, and both D1R and D2R. Data are shown as the mean ± SD in the medial (**a**), intermediate (**b**), and lateral (**c**) divisions

The distribution of D1R- and D2R-expressing neurons showed a distinctive pattern in the tri-laminar part (Fig. 4). The medial division contained many D1R-expressing neurons but far fewer D2R-expressing neurons, consistent with previous observations (Gangarossa et al. 2013). In contrast, the intermediate division contained a number of D2R-expressing neurons, but few D1R-expressing neurons. In the lateral division, both D1R- and D2R-expressing neurons were uniformly distributed with similar densities. Quantitative analysis in three animals indicated that the proportion of D1R-expressing neurons in the medial, intermediate, and lateral division was 88.6 ± 8.2% (651 total cells), 14.7 ± 3.8% (1025 cells), and 49.3 ± 4.5% (873 cells), respectively (Fig. 5), with statistically significant differences among each of these divisions (Tukey–Kramer test, *n* = 3 animals; *p* < 1.0 × 10^−7^ in the comparison between the medial and intermediate divisions, *p* = 3.0 × 10^−6^ between the intermediate and lateral divisions, *p* = 1.5 × 10^−6^ between the medial and lateral divisions). In contrast, the proportion of D2R-expressing neurons in the medial, intermediate, and lateral division was 11.1 ± 8.1%, 83.1 ± 3.5%, and 48.1 ± 5.0%, respectively, with statistically significant differences among each of these divisions (Tukey–Kramer test, *n* = 3 animals; *p* < 1.0 × 10^−7^ in the comparison between the medial and intermediate divisions, *p* = 1.3 × 10^−6^ between the intermediate and lateral divisions, *p* = 1.0 × 10^−6^ between the medial and lateral divisions). The proportion of neurons double-labeled for D1R- and D2R was very low; that in the medial, intermediate, and lateral divisions was 0.3 ± 1.1%, 2.2 ± 2.4%, and 2.6 ± 3.5%, respectively.

**Fig. 6:**
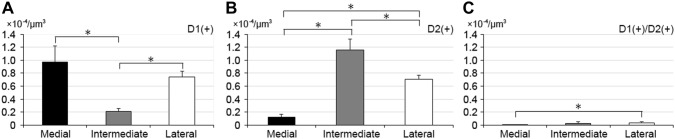
Uneven distributions of striatal neurons that differ by location within the tri-laminar part. The cell density per unit volume of D1R-, D2R-, and D1R/D2R-double expressing neurons is shown in **a**–**c**, respectively (Asterisks, *p* < 0.05, Tukey–Kramer test)

When the density of labeled neurons per unit volume was compared among the three divisions, the density of D1R-expressing neurons (Fig. 6a) was lowest in the intermediate division (Tukey–Kramer test, *n* = 3 animals; *p* = 0.0024 in the comparison between the medial and intermediate divisions, *p* = 0.014 between the intermediate and lateral divisions), whereas it was comparable between medial and lateral divisions (*p* = 0.24). In contrast, the density of D2R-expressing neurons (Fig. 6b) was highest in the intermediate division (Tukey–Kramer test, *n* = 3 animals; *p* = 5.1 × 10^−5^ in the comparison between the medial and intermediate divisions, *p* = 0.0046 between the intermediate and lateral divisions). The density of D2R-expressing neurons was also significantly different between the medial and lateral divisions (*p* = 0.0013). Though much lower in density, neurons double-labeled for D1R and D2R (Fig. 6c) were encountered more often in the lateral division than medial division (*p* = 0.016). These findings suggest that each division in the tri-laminar part consists of a differential proportion of direct and indirect pathway neurons.

### Distribution of interneurons

**Fig. 7:**
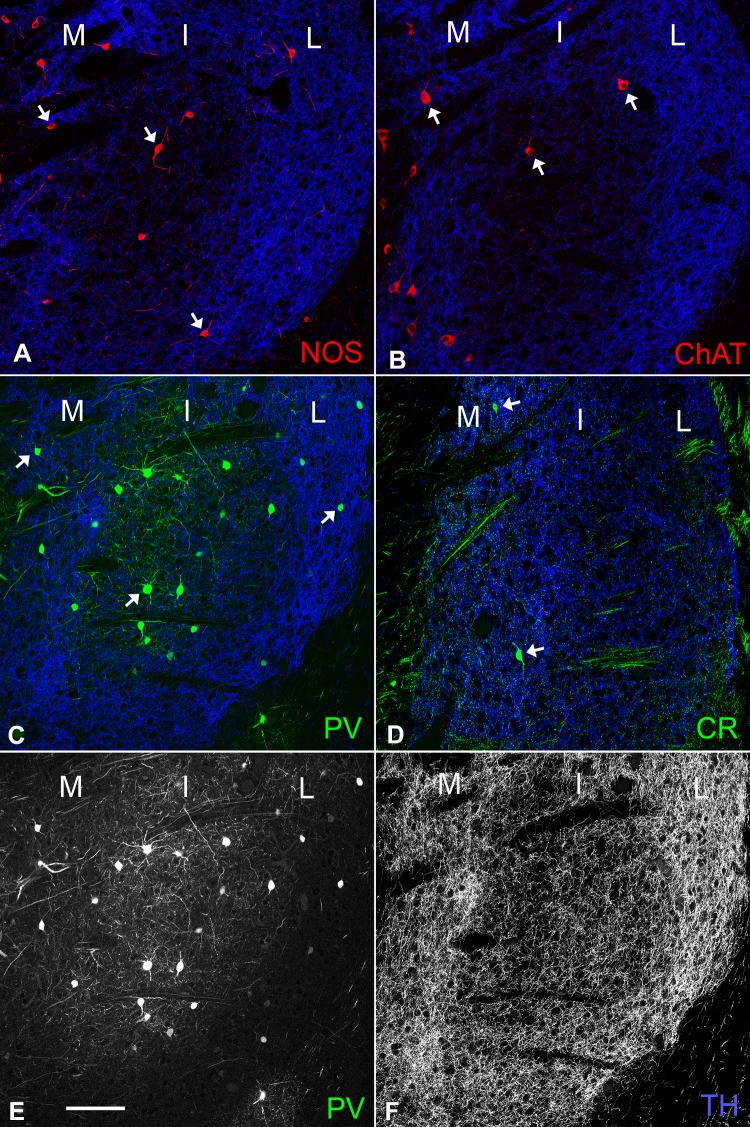
Distribution of the four types of interneurons in the tri-laminar part. In the pseudocolor images (**a**–**d**), immunostaining for NOS and ChAT is shown in red in (**a**) and (**b**), for PV and CR in green in (**c**) and (**d**), and for TH in blue throughout the images. Arrows indicate labeled interneurons in each immunostaining image. PV and TH immunoreactivities in (**c**) are shown separately in (**e**) and (**f**). Scale bar 100 µm

**Fig. 8:**
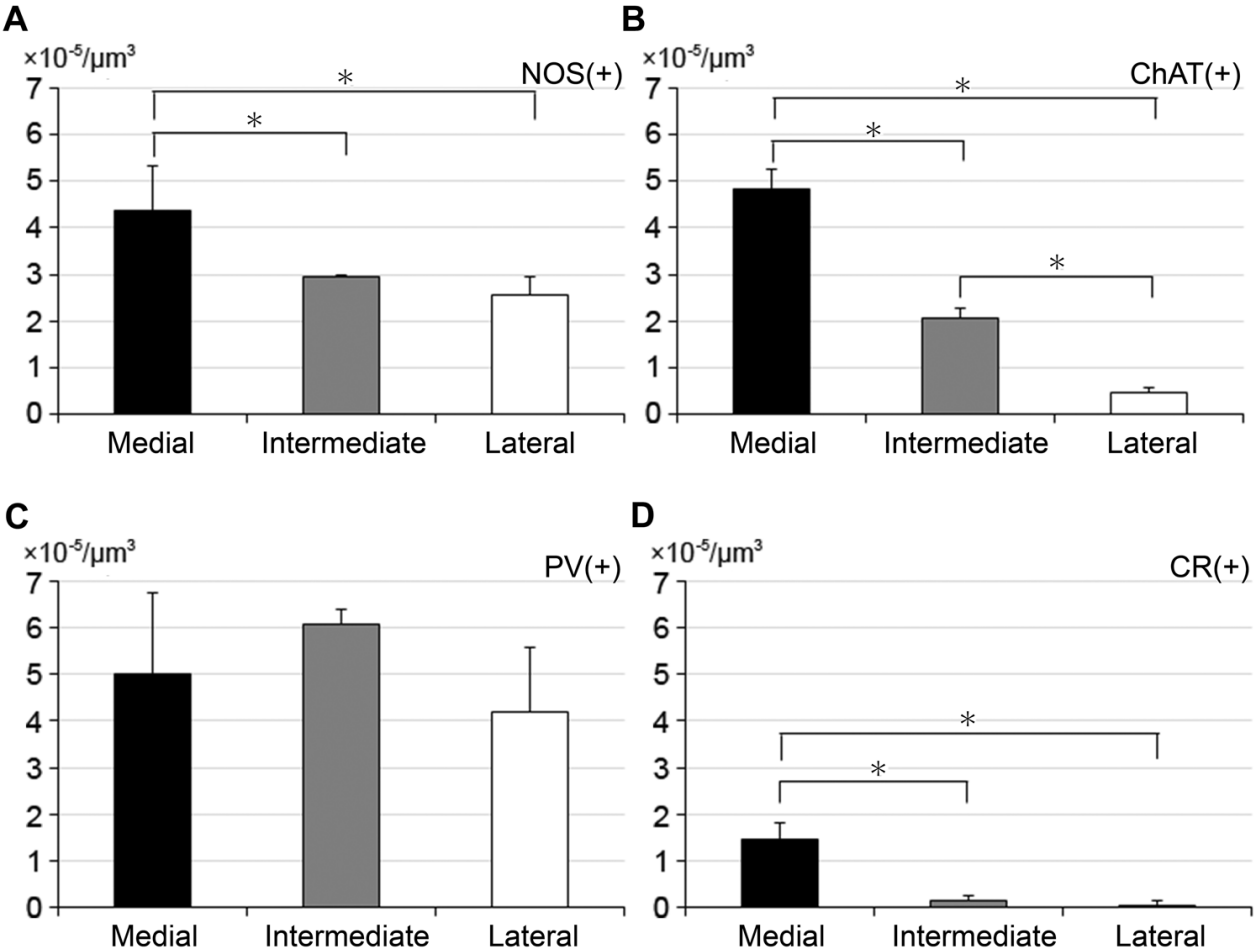
Uneven distribution of striatal interneurons within the tri-laminar part. The cell density per unit volume of NOS-, ChAT-, PV-, and CR-positive interneurons are shown in (**a**–**d**), respectively (Asterisks, *p* < 0.05 in Tukey–Kramer test)

All NOS-, ChAT-, PV-, and CR-immunoreactive neurons were identified in each division of the tri-laminar part (Fig. 7), although CR-positive neurons were rarely observed, as reported in a previous study (Gangarossa et al. 2013). Quantitative analysis of cell density per unit volume showed that NOS-, ChAT- and CR-positive neurons possessed the highest density in the medial division (Fig. 8a, b, d; Tukey–Kramer test, *n* = 4 animals; NOS, *p* = 0.014 in the comparison between the medial and intermediate divisions, *p* = 0.0029 between the medial and lateral divisions; ChAT, *p* = 3.3 × 10^−6^ between the medial and intermediate divisions, *p* = 1.0 × 10^−7^ between the medial and lateral divisions; CR, *p* = 2.6 × 10^−4^ between the medial and intermediate divisions, *p* = 1.6 × 10^−4^ between the medial and lateral divisions). In addition, the density of ChAT-positive neurons differed significantly between the intermediate and lateral divisions (*p* = 2.3 × 10^−4^). In contrast, PV-positive somata were distributed in all three divisions at a relatively constant, high density (Fig. 8c). However, PV immunoreactivity stood out in the intermediate division due to dense arbors of dendrites arising from large somata (Fig. 7e), which was contrasted by the weak TH immunoreactivity in the intermediate division (Fig. 7f).

## Discussion

Current knowledge on the structure and function of the rodent striatum depends largely on analyses of the rostral three-quarters of the striatum. However, the present study has demonstrated that the caudal striatum contrasts itself with the rostral striatum in many aspects. For example, this region does not have typical striosome/matrix compartmentalization as seen in the rostral striatum, but consists of three distinct divisions, and its intermediate division is innervated only sparsely by dopaminergic axons. These features indicate the necessity of understanding the caudal striatum from a distinct viewpoint from the conventional one for the stratum.

### Definition of the three divisions of the caudal striatum

In our previous study, we characterized three divisions of the caudal striatum on a basis of contrasting labeling patterns for SP and Enk (Miyamoto et al. 2018). The present quantitative analysis of the labeling intensity in both SP and Enk immunohistochemistry further demonstrates the validity of dividing the caudal striatum into three areas. Therefore, apparent change in SP immunoreactivity across the medial to intermediate border and that in Enk immunoreactivity across the intermediate to lateral border facilitated the procedure for defining three divisions in the acquired confocal images. However, this observation-based procedure may have led to some fluctuation in the positions of borders, which further may have resulted in some measurement errors in the cell density analysis. To address this issue, we averaged the data obtained from multiple measurements that were expected to balance and minimize measurement errors. In fact, the comparison of cell density of D1R/D2R-expressing projection neurons (Fig. 6) and three types of interneurons (Fig. 8) showed marked differences between divisions with robust statistical significance. This suggests that the general conclusions will not change after fine adjustments of the positions of borders.

### Differential immunoreactivities in the tri-laminar part

Classification of the tri-laminar part into medial, intermediate, and lateral divisions is based on differential immunoreactivities to SP and Enk (Miyamoto et al. 2018), both of which are contained in axon terminals but not in somata (Lee et al. 1997; Shigematsu et al. 2008). In the present study, we demonstrated that the composition of resident cells, which can be the postsynaptic recipient of the SP and Enk positive axon terminals, is different among the divisions defined by SP/Enk immunoreactivity.

Previous studies in rats have repeatedly described a narrow zone with intense SP labeling at the boundary between the striatum and GP, which is termed the “marginal division (MrD)” (Shu et al. 1988, 1990) and is observed in cat, monkey, and humans (Shu et al. 1999). Recent studies using BAC transgenic mice that express enhanced green fluorescence protein under the control of *Drd1a* or *Drd2* promoters identified a similar zone containing almost exclusively *Drd1a*-expressing neurons, and the region was termed the “D2R/A2aR-expressing MSNs-poor zone” (Gangarossa et al. 2013). The immunohistochemical properties of the medial division shown here in both wild-type and transgenic mice are consistent with these observations. On the other hand, neighboring regions consisting of intermediate and lateral divisions showed characteristic immunohistochemical features that have not been previously investigated. Comparative studies of the tri-laminar part defined by objective borders will accelerate understanding of the caudal striatum, which has drawn much attention recently (Menegas et al. 2015, 2017, 2018; Guo et al. 2018).

Intense labeling of SP in the medial division can be explained by high proportions (nearly 90%) of D1R-expressing neurons in the same division. That is because SP is contained in axon terminals of dMSNs that express D1R and because local axon collaterals of striatal MSNs ramify compactly in their surrounding regions (Wilson and Groves 1980; Kawaguchi et al. 1989). This explanation is also applicable to striosomal neurons in the rostral striatum where striosomes showing intense SP immunoreactivity contain higher proportions of D1R neurons (Miyamoto et al. 2018).

However, this rather simplistic relationship does not apply to the intermediate division. Though this region contains higher proportions (~ 83%) of D2R-expressing neurons, Enk labeling was weaker than in the lateral division where D2R-expressing neurons comprised only half of MSNs. One possibility, albeit speculative, is that neurons in the intermediate division might not form local collaterals as densely as in the medial or lateral divisions. Interestingly, this appears to be applicable to the most lateral portion of the rostral striatum. This part is devoid of striosome/matrix compartmentalization, and not only SP but also Enk immunoreactivities are maintained at low levels there, though the proportion of D2R-expressing neurons in this striosome-free space is as high as 65% (Miyamoto et al. 2018). These labeling patterns suggest site-specific heterogeneity in the degree of local interconnectivity among MSNs.

The medial division is clearly demarcated from the surrounding region in SP labeling, which raises the possibility that the medial division can be seen as a striosome-related structure, because one of the remarkable properties of compartmentalization in the striatum is its site-dependent diversity (Miyamoto et al. 2018). Conventional striosomes have common structural features such that they have round, oval, or elongated shapes with an occasional bifurcation or intervening thin bridges and have relative constant transverse diameter, forming a labyrinth (Graybiel et al. 1981; Desban et al. 1993; Tajima and Fukuda 2013). In contrast, the medial division has a slab-like form of much larger size. Another differential feature is that the medial division of the tri-laminar part contains a distinctive type of large-sized GABAergic neurons that are never observed in conventional striosomes throughout the striatum (data will be shown elsewhere). It can at least be said that compartmentalization of the striatum is more complicated than has been previously thought, and it remains an open question whether the medial division is a specialized part of striosome/matrix compartmentalization.

### Distributions of interneurons in the tri-laminar part

Cholinergic and three types of GABAergic interneurons are interspersed within the mouse striatum (Kawaguchi et al. 1995). These interneurons constitute only 5% of all striatal neurons, but play important roles in the control of MSNs (Tepper et al. 2010; Gittis and Kreitzer 2012). Each interneuron has different morphological and electrophysiological properties and tends to exhibit a somewhat biased distribution pattern (Kawaguchi et al. 1995). In the caudal striatum, the presence of representative interneurons has been confirmed in the area corresponding to the medial part of the tri-laminar structure (Gangarossa et al. 2013), and the present results extended these observations over all divisions of the tri-laminar part.

As cholinergic neurons have NK-1 receptors (Elde et al. 1990; Gerfen 1991) and are depolarized by bath-applied SP in acute slice preparations (Aosaki and Kawaguchi 1996), regulatory activities of cholinergic neurons on MSNs will be induced through SP originating from D1R-expressing MSNs. The present study demonstrated that the medial division of the tri-laminar part, which is identified as an SP-rich zone, is further characterized by a higher cell density of both ChAT-positive neurons and D1R-expressing cells. These observations indicate that the above-mentioned SP- and NK-1 receptor-mediated regulatory mechanisms will be especially important in the medial division of the tri-laminar part. Furthermore, the cell density of NOS- and CR-positive neurons is also highest in the medial division, suggesting that MSNs in the medial division may be under elaborate control of various interneurons.

PV-positive interneurons are distributed throughout the tri-laminar part with relatively high cell density in all divisions. This suggests pivotal roles of PV/GABAergic interneurons in the regulation of principal cell populations, which is in agreement with their properties wherein they preferentially synapse onto somata of MSNs over any other input (Kita et al. 1990), thereby controlling spiking activities of MSNs as fast-spiking neurons (Kawaguchi 1993; Koós and Tepper 2002). However, the relative abundance of PV-labeled dendritic nets, which are interconnected through gap junctions in the striatum of the cat (Fukuda 2009) and mouse (unpublished observations), in the intermediate division, together with the contrasting low TH labeling, suggests regional differences in the mode of regulation of MSN activities among these three divisions.

## Functional implications

The striatum receives inputs from the entire neocortex with a topographical connectivity that has been extensively studied in projectome analyses (Hintiryan et al. 2016; Hunnicutt et al. 2016). However, information available regarding the source of cortical afferents to the caudal striatum is rather limited, and the detailed positions of cortical afferents inside the caudal striatum, such as distinction between the dorsal and ventral halves of the caudal striatum, have not necessarily been described in previous studies. In our recent study, we demonstrated that the primary targets of the afferents from the primary auditory cortex and agranular insula were the intermediate and lateral divisions of the tri-laminar part, respectively (Miyamoto et al. 2018). These connectivity patterns are consistent with findings in a connectome study (Hintiryan et al. 2016) and are in line with a retrograde labeling study showing that the most caudal portion of the rat striatum, albeit the sites of injection appear to cover broad regions of the caudal striatum, is innervated from the auditory, visual, somatosensory, and gustatory cortex (Jiang and Kim 2018). Recent progress in mapping the auditory cortex using optical recordings in vivo has been leading to revision of the architecture of the auditory cortex (Takemoto et al. 2014). Thus, combining updated knowledge about the auditory system with finer tools for structural analysis will illuminate the function of the tri-laminar part.

A possible relationship between the tri-laminar part and auditory processing is also supported by the distribution of thalamic inputs from the medial division of the medial geniculate nucleus in the tri-laminar part (LeDoux et al. 1985). This anatomical finding is consistent with the distribution of physiologically recorded units with short latency to auditory stimuli detected in the regions covering both the tri-laminar part, especially in its lateral division, and the neighboring lateral amygdaloid nucleus (Bordi and LeDoux 1992). Response properties of neurons in the caudal striatum are reported to be similar to those in the lateral amygdala, suggesting the possible involvement of the tri-laminar part in rapid behavioral responses, as in the amygdala (Romanski and LeDoux 1992). Interestingly, the lateral division of the tri-laminar part receives cortical inputs from the agranular insula (Miyamoto et al. 2018), which is reminiscent of the convergence of both subcortical and cortical information to the emotional center, leading to differential aspects of fear conditioning (Romanski and LeDoux 1992). Alternatively, the agranular insula is also suggested to be involved in reward prediction after sensory cues (Kesner and Gilbert 2007), and the flow of sensory information from the agranular insula through lateral division of the tri-laminar part might constitute a parallel loop of the basal ganglia for sensory-evoked reward prediction in which rich innervation of dopamine fibers in the lateral division (Fig. 6f) and balanced activities of the two types of striatal neurons expressing D1R or D2R (Fig. 4c) may play roles.
